# Incidence of Kidney Stones After Metabolic and Bariatric Surgery—Data from the Scandinavian Obesity Surgery Registry

**DOI:** 10.1007/s11695-023-06561-y

**Published:** 2023-03-31

**Authors:** Anna Laurenius, Magnus Sundbom, Johan Ottosson, Erik Näslund, Erik Stenberg

**Affiliations:** 1 Institute of Clinical Sciences, Department of Surgery, Sahlgrenska Academy, University of Gothenburg, Vita stråket 11, S-413 45 Gothenburg, Sweden; 2 Department of Surgical Sciences, Uppsala University, Uppsala, Sweden; 3 Department of Surgery, Faculty of Health and Medicine, Örebro University, Örebro, Sweden; 4 Division of Surgery, Department of Clinical Sciences, Danderyd Hospital, Danderyd, Sweden; 5 Karolinska Institutet, Stockholm, Sweden

**Keywords:** Bariatric surgery, Sleeve gastrectomy, Gastric bypass, Biliopancreatic diversion, Hyperoxaluria, Kidney stones, Nephrolithiasis

## Abstract

**Purpose:**

Obesity is associated with increased incidence of kidney stones, a risk further increased by metabolic and bariatric surgery, particularly after procedures with a malabsorptive component. However, there is a paucity in reports on baseline risk factor and on larger population-based cohorts. The objective was to evaluate incidence and risk factors for kidney stones after bariatric surgery by comparing them to an age-, sex-, and geographically matched cohort from the normal population.

**Material and Methods:**

Patients operated with primary Roux-en-Y gastric bypass (RYGB), sleeve gastrectomy (SG), or biliopancreatic diversion with duodenal switch (BPD-DS) from 2007 until 2017 within the Scandinavian Obesity Surgery registry were matched 1:10 to controls from the normal population. Hospital admission or outpatient visits due to kidney stones registered in the National Patient Registry were considered as endpoint.

**Results:**

The study included 58,366 surgical patients (mean age 41.0±11.1, BMI 42.0±5.68, 76% women) with median follow-up time 5.0 [IQR 2.9–7.0] years and 583,660 controls. All surgical procedures were associated with a significantly increased risk for kidney stones (RYGB, HR 6.16, [95% CI 5.37–7.06]; SG, HR 6.33, [95% CI 3.57–11.25]; BPD/DS, HR 10.16, [95% CI 2.94–35.09]). Higher age, type 2 diabetes hypertension at baseline, and a preoperative history of kidney stones were risk factors for having a postoperative diagnosis of kidney stones.

**Conclusion:**

Primary RYGB, SG, and BPD/DS were all associated with a more than sixfold increased risk for postoperative kidney stones. The risk increased with advancing age, two common obesity-related conditions, and among patients with preoperative history of kidney stones.

**Graphical Abstract:**

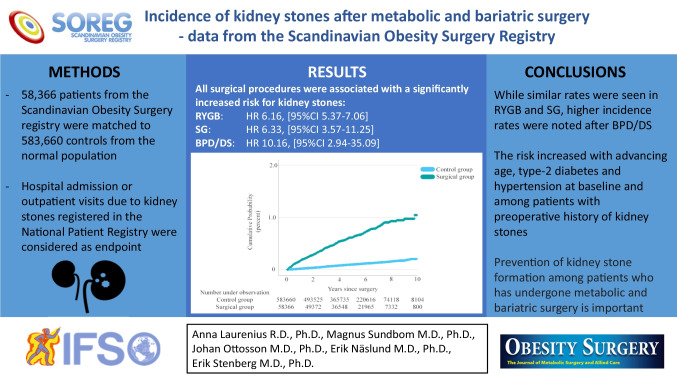

## Introduction

Metabolic and bariatric surgery (MBS) is an effective and safe treatment for individuals with severe obesity [1]; still, these surgeries come with a risk of various complications [2]. During the 1970s and 1980s, attention was drawn to the problem of kidney stones in patients having jejunoileal bypass for obesity [3], but kidney stones also occur after modern MBS [4]. In addition to acute abdominal pain, kidney stones can lead to hypertension, chronic kidney disease, and end-stage kidney disease [5].

Obesity itself is a known risk factor for kidney stones with an incidence reported to be between 4.3 and 4.6% [6, 7]; however, the risk increases after MBS, especially in procedures with a malabsorptive component [8–11]. The frequency does not appear to be increased after procedures considered to be of more restrictive character, where the incidence rate has been reported to be 3.7% [9], while an incidence rate of 7.7–8.1% has been seen after Roux-en-Y gastric bypass (RYGB) [6, 9]. In a systematic review and a meta-analysis, the relative risk for kidney stones was 0.37 and 0.29, respectively, for purely restrictive surgery, including sleeve gastrectomy (SG), versus 1.73 and 1.79 for RYGB [10, 11]. Three studies that specifically compared SG with RYGB all showed a higher risk of kidney stones after RYGB [8, 9, 12]. The incidence of kidney stones after MBS in large patient samples is unknown, and there is a paucity in studies assessing baseline factors associated with postoperative kidney stones.

The aim of the study was to evaluate the incidence of kidney stones in a large nationwide population undergoing the three most common primary MBS compared to an age-, sex-, and geographically matched control group from the normal population, and to evaluate factors associated with a postoperative diagnosis of kidney stones.

## Materials and Methods

This observational, matched cohort study was based on data from the Scandinavian Obesity Surgery registry (SOReg), a national quality register that started in 2007. SOReg covers virtually all MBS procedures in Sweden and has high internal validity [13]. By using the national identification numbers (unique to all Swedish citizens), the SOReg database was linked to the National Patient Register (NPR) for retrieving diagnoses of inpatient and outpatient care, and the total population registry, having complete coverage of mortality, for achieving correct observation time. The NPR includes nearly 100% of all hospital admissions in public health care, while the outpatient component, started in 2001, covers about 95% of outpatient visits in specialized health care [14].

All adults (≥18 years) with a preoperative BMI >30 kg/m^2^ (MBS is recommended in Sweden for BMI greater than 30 with poorly controlled T2D and BMI greater than 35 without comorbid disease according to the recently updated guidelines), operated with a primary RYGB, SG, or biliopancreatic diversion with a duodenal switch (BPD-DS) from Jan 2007 until Sept 2017, were considered for inclusion. Patients who underwent a SG as a first step before a secondary planned MBS were excluded.

### Definitions

Comorbidity was defined as an obesity-related condition, e.g., hypertension, type 2 diabetes (T2D), dyslipidemia, and depression, requiring active pharmacological treatment or continuous positive airway pressure treatment for sleep apnea. The outcome, kidney stones, was defined as hospital admission or outpatient visits in an acute care facility or specialized care with the diagnosis kidney stones (ICD-10: N20.0-N20.9).

### Statistics

Continuous variables assuming a normal distribution are presented as the mean ± standard deviation (SD), while categorical variables are presented as numbers (*n*) and proportions (%). Patients were followed until diagnosed kidney stone, death, emigration, or 31 December 2017 which ever came first. The Kaplan-Meier method was used to visualize cumulative probability (1-Kaplan-Meier estimate), with incidences evaluated as incidence rates (IR)/1000 person-years. The risk for kidney stone formation was estimated using Cox regression with hazard ratios (HRs) and 95% confidence intervals (CIs) as measures of association. Preoperative variables were assessed as risk factors for postoperative kidney stones among the operated group using unadjusted Cox regression and further in a multivariable model including all preoperative variables. A second Cox regression model including postoperative weight reduction at 1 year after surgery was performed adjusted for all preoperative variables. IBM SPSS version 27 (IBM, Armonk, NY, US) and R version 4.1.3 (R Core Team, Vienna, Austria) were used for statistical analyses.

### Ethics

The study was approved by the Swedish ethical review authority (Ref nr 2020-03005) and conducted in accordance with the standards of the 1964 Helsinki Declaration and its later amendments.

## Results

During the study period, 58,366 patients who underwent a primary RYGB, SG, or BPD/DS were identified from the Scandinavian Obesity Surgery registry (SOReg). They were matched on age, sex, and place of residence (county) to 583,660 controls from the normal population, identified from the National Patient Register (NPR). Overall median follow-up time for the MBS patients and controls were 5.0 (IQR 2.9–7.0) years, with 5.5 (3.6–7.2) years for RYGB and their controls, 2.0 (1.1–3.0) years for SG and their controls, and 4.8 (2.7–6.8) years for BPD/DS and 4.9 (2.9–7.0) years for their controls Table 1.

**Table 1:** Baseline characteristics

	Entire group except controls	RYGB	SG	BPD/DS	Control group
Number of patients	58,366	51,152	6781	433	583,660
Age	41.0 ± 11.1	40.9 ± 11.2	41.2 ± 10.7	37.8 ± 10.8	41.0 ± 11.1
BMI	42.0 ± 5.68	42.3 ± 5.42	39.4 ± 5.70	56.5 ± 6.78	Unknown
Sex					
Women	44,459 (76.2%)	38,719 (75.7%)	5488 (80.9%)	252 (58.2%)	444,590 (76.2%)
Men	13,907 (23.8%)	12,433 (24.3%)	1293 (19.1%)	181 (41.8%)	139,070 (23.8%)
Comorbidity					
Hypertension	14,459 (19.4%)	13,012 (25.4%)	1316 (19.4%)	131 (30.3%)	51,134 (8.8%)
Type 2 diabetes	7978 (13.7%)	7323 (14.3%)	577 (8.5%)	78 (18.0%)	13,120 (2.2%)
Dyslipidemia	5558 (9.5%)	5053 (9.9%)	461 (6.8%)	44 (10.2%)	24,457 (3.7%)
Depression	8788 (15.1%)	7582 (14.8%)	1132 (16.7%)	74 (17.1%)	67,029 (11.5%)
Sleep apnea	5650 (9.7%)	5103 (10.0%)	435 (6.4%)	112 (25.9%)	Unknown
Previous kidney stones	190 (0.3%)	175 (0.3%)	11 (0.2%)	4 (0.9%)	543 (0.1%)

*RYGB* = Roux-en-Y gastric bypass; *SG* = sleeve gastrectomy; *BPD/DS* = biliopancreatic diversion with duodenal switch

Data on BMI and sleep apnea could not be obtained for the control group; otherwise, there were no missing data for any of the baseline characteristics

### Incidence of Kidney Stones

During follow-up, 356 patients in the operated group (IR 1.22 [1.10–1.36] per 1000 person-years; HR 6.20 [5.43–7.08], *p*<0.001) had at least one episode with kidney stone, compared to 575 patients in the control group (IR 0.20 [0.18–0.21] per 1000 person-years). The incidence of kidney stones was more than sixfold higher among patients operated with RYGB and SG (IR 1.21 [1.09–1.35] per 1000 person-years; HR 6.16 [5.37–7.06]) and (IR 1.28 [0.77–1.99] per 1000 person-years; HR 6.33 [3.57–11.25, *p*<0.001 for both) compared to their respective controls (IR 0.20 [0.18–0.21] per 1000 person-years and IR 0.20 [0.14–0.29] per 1000 person-years). While similar rates were seen in RYGB and SG, higher incidence rates were noted after BPD/DS (IR 2.35 [0.47–4.71] per 1000 person-years, *vs.* 0.23 [0.05–0.46] per 1000 person-years; HR 10.16 [2.94–35.09] compared to their controls, *p*<0.001).

### Risk Factors for Kidney Stones

Higher age, T2D and hypertension at baseline, and previous kidney stones were all associated with a higher risk for a postoperative diagnosis of kidney stones in the multivariable analysis (Table 2).

**Table 2:** Evaluation of risk factors associated with postoperative kidney stones

	Univariable analysis	Multivariable analysis	
	OR (95% CI)	OR (95% CI)	*P*
Age			
<30	Reference	Reference	Ref
30–39	1.58 (0.94–2.65)	1.37 (0.82–2.31)	0.231
40–49	3.41 (2.13–5.47)	2.39 (1.47–3.88)	<0.001
50–59	5.38 (3.35–8.64)	2.83 (1.71–4.68)	<0.001
>60	8.46 (4.98–14.39)	3.60 (2.03–6.36)	<0.001
BMI, at baseline	1.00 (0.99–1.02)	1.01 (0.99–1.03)	0.249
Sex			
Women	Reference	Reference	Ref
Men	1.78 (1.43–2.21)	1.21 (0.96–1.53)	0.107
Comorbidity			
Hypertension	3.46 (2.81–4.27)	1.70 (1.32–2.19)	<0.001
Type 2 diabetes	3.98 (3.22–4.92)	2.18 (1.70–2.79)	<0.001
Dyslipidemia	2.56 (1.99–3.31)	0.94 (0.71–1.25)	0.667
Depression	1.34 (1.03–1.75)	1.27 (0.97–1.66)	0.084
Sleep apnea	2.00 (1.52–2.63)	1.07 (0.80–1.43)	0.658
Previous kidney stones	24.1 (15.76–36.72)	12.16 (7.87–18.79)	<0.001
Surgical Method			
RYGB	Reference	Reference	Ref
SG	0.89 (0.56–1.43)	1.04 (0.65–1.66)	0.877
BPD/DS	1.92 (0.80–4.66)	1.33 (0.53–3.36)	0.542

*OR =* odds ratio; *95% CI =* 95% confidence interval; *Ref =* Reference; *RYGB =* Roux-en-Y gastric bypass; *SG =* Sleeve gastrectomy; *BPD/DS =* biliopancreatic diversion with duodenal switch

Postoperative weight loss at 1 year was reported for 46,393 patients (79.5%), with a mean TWL of 31.5 ± 7.9%, BMI loss 13.4 ± 4.1 kg/m^2^, and EBMIL 81.3 ± 23.4%. The total weight loss was inversely associated with kidney stone formation (unadjusted—HR 0.98 [0.97–0.99] per %TWL, *p*=0.008), but not statistically significantly so after adjustment for other covariates (adjusted—HR 1.01 [1.00–1.03], *p*=0.157).

## Discussion

In this large registry study, the incidence of kidney stones after primary MBS was increased by more than sixfold compared to matched controls from the normal population The highest incidence per 1000 person-years was seen after BPD-DS (2.35 cases), while RYGB and SG had similar incidences (1.21 and 1.28, respectively), all in comparison to 0.2 in the normal population. Risk factors were higher age, hypertension and T2D at baseline, and in particular previous kidney stones. We could not demonstrate that preoperative BMI, nor degree of postoperative weight loss, was associated with symptomatic kidney stones.

### Overall Increase

The relative risk for kidney stones on pooled data was 1.22 (95% CI 0.63–2.35) in the meta-analysis from Thongprayoon [11] with a 1.73-fold risk increase after RYGB, which is lower than the 6–10-fold risk increase in the present study. The higher risk in the present study is likely to be attributed to the control group, here representing the normal population and in the meta-analysis a non-operated group of individuals with BMI >35 kg/m^2^.

**Fig. 1:**
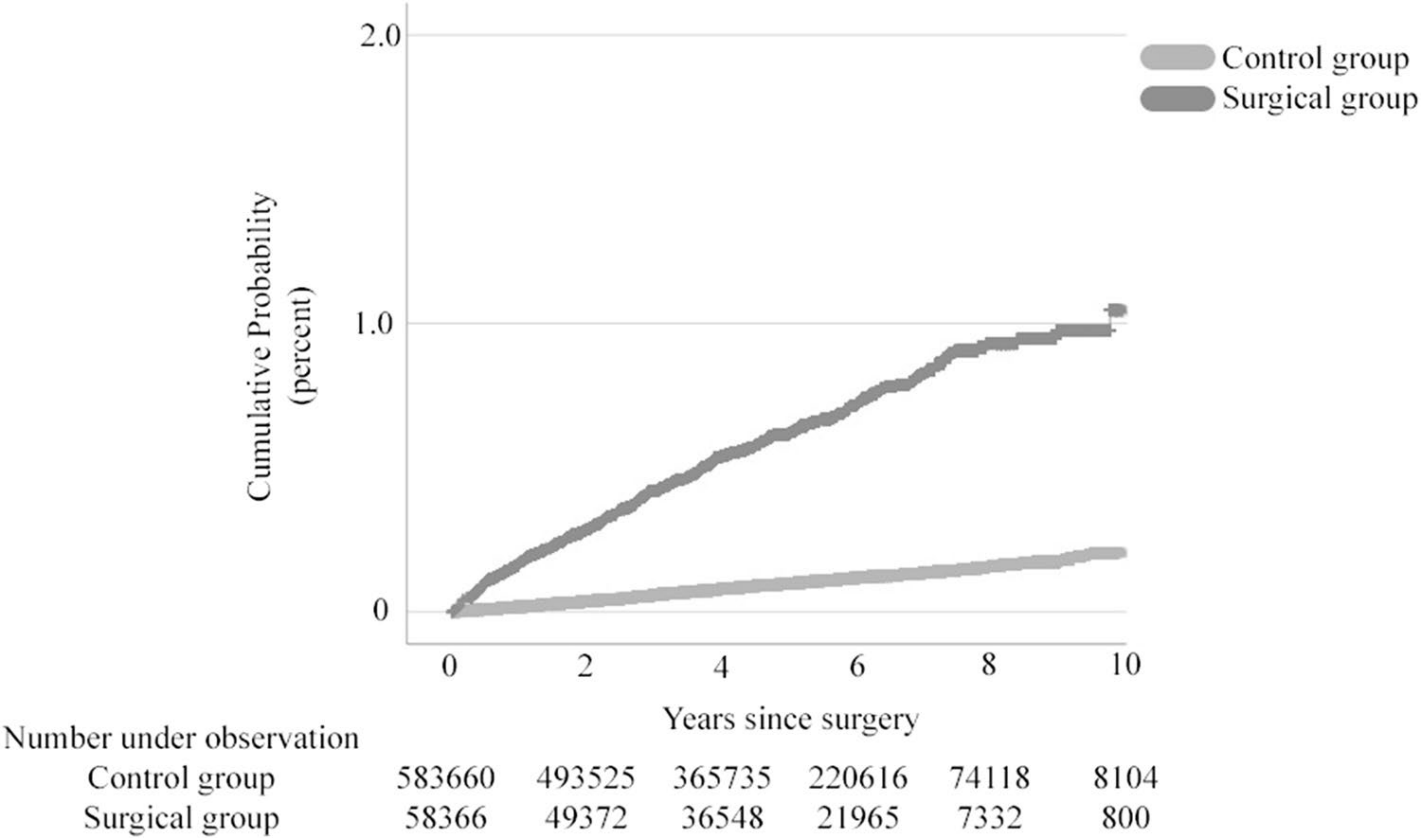
Cumulative probability for kidney stones for patients operated with primary MBS compared to matched non-operated controls

The cumulative increased risk for kidney stones during the 10-year study period (demonstrated in Fig. 1) is probably due to physiological factors and not continuous weight loss, as we failed to see any differences at the first postoperative year, when most of the weight loss has occurred. We, however, noticed an age-dependent stepwise increase in the diagnosis of kidney stones, just as in non-operated controls.

### Specific Procedures

The pathophysiology behind kidney stone formation is fat malabsorption, as it causes increased binding of dietary calcium to free fatty acids, thus reducing the available amount of calcium to immobilize dietary oxalate. Furthermore, the presence of unabsorbed bile salts and fatty acids in the colon increases colonic permeability, which raises intestinal oxalate absorption, leading to hyperoxaluria and increased risk of oxalate stone formation [15–17].

The highest risk for symptomatic kidney stones was seen after BPD/DS which probably depends on several factors. Firstly, the procedure results in vast physiological changes, including the above-mentioned increase of bile salts and fatty acids in the colon, increasing the risk for oxalate stone formation. Furthermore, as the calcium homeostasis is under stress due to low vitamin D levels, the overall capacity to take care of oxalate can be reduced. Secondly, an altered dietary pattern due to frequent problems with diarrhea, including a relative hypovolemia, may both induce the formation of kidney stones and impair their natural elimination. Thirdly, a higher proportion of BPD/DS patients were men (41.8% *vs.* 24–19% in the two other procedures). Although sex was not considered a significant risk factor in the multivariable analysis, higher risk has been reported among men compared to women [18].

What discriminates our study from most other studies is that the incidence rate between RYGB and SG was similar. One explanation for this difference could be that many other studies have published data of SG pooled with restrictive surgery such as vertical banded gastroplasty or gastric banding. These studies has stated that SG and restrictive surgery has similar [7, 19] or lower incidence compared to RYGB or surgery which includes a component of malabsorption [11]. However, three studies have compared the incidence specifically between SG and RYGB, all showed a higher incidence of kidney stones after RYGB [8, 9, 12], but two of the studies had lower incidence of T2D in the SG group [9, 12] and only one study reported hypertension [12], factors associated with increased risk for kidney stones in the present study.

Our finding with a similar incidence of kidney stones between RYGB and SG is intriguing. Assuming that SG is a restrictive method may be incorrect which also has been discussed in several reviews [20, 21]. Although we do not yet have a full understanding of the mechanisms of action after SG, it has been shown that, similar to RYGB, factors other than restriction, such as vagal and hypothalamic activity, gastrointestinal hormone responses, energy expenditure alteration, and changes in bile acid synthesis and microbiota composition also contribute to the weight change and metabolic improvements seen after the procedure [21]. It has been claimed that after RYGB, the malabsorptive component is small, but fecal fat excretion has shown to be increased compared to preoperative levels in the short term after surgery [16, 22]. However, we have not been able to find any study that examined the difference in fat excretion or gut microbiota between SG and RYGB. In contrast, steatorrhea and hyperoxaluria were common in patients with obesity before and after RYGB hyperoxaluria correlated with steatorrhea and has been proposed to be associated with excess fatty acids in the intestinal lumen. Furthermore, before and after RYGB, high oxalate intake seems to contribute to the severity of hyperoxaluria [23]. Previous studies have reported lower levels of hyperoxaluria after SG compared to RYGB [8, 24, 25].

### Preventing the Formation of Kidney Stones

Besides comparing the long-term effects on weight and comorbidity and overall risk of complications, the increased risk of kidney stones must be considered during preoperative counseling. Although not being a difficult clinical problem in most cases, prevention of kidney stone formation among patients who has undergone MBS is important. Several nutritional interventions may be needed. A reduction in oxalate intake and avoidance of gram doses of vitamin C supplementation, which is converted to oxalate, should be recommended as well as reducing fat intake. This is because patients with long-term steatorrhea have an increased excretion of oxalate via the kidneys and under normal circumstances, dietary calcium binds to the oxalate to a salt that cannot be absorbed. In steatorrhea, calcium is instead bound to the fat, which is why oxalate can be more easily absorbed. Calcium and vitamin D should be increased as well as fluid intake and finally lactic acid bacteria that can increase the breakdown of oxalate in the intestine [10, 16, 26].

In the group that underwent surgery, an average of 0.3% had kidney stones preoperatively; however, this is not listed as a contraindication for MBS in national guidelines. Based on current study and previous literature, SG or RYGB would be the method of choice in patients with pronounced problems with kidney stones BPD/DS should be avoided.

### Limitations

While this study represents a large cohort of patients with a matched control group from the normal population and inclusion of high-quality data from several national databases [13, 14], there are limitations that must be acknowledged. Firstly, the definition of kidney stones used in this model was defined on contacts with specialized care. While most patients presenting with kidney stones are expected to be in contact with an emergency department or followed at a specialized clinic, some patients with limited symptoms, handled in primary care, would be missed using this definition. This may not be so important as we probably have captured all severe cases. Secondly, due to long delivery time for our linked data from the official national registries, the follow-up was limited to the end of 2017. For the SG group, more recent data with improved follow-up time would have been valuable. However, based on the variable concerning previous kidney stones (0.2% *vs.* 0.3% for RYGB), the risk of selection bias seems minute. Furthermore, no data on the type of kidney stone could be obtained via the registers, but based on a report from Mishra et al., oxalate stones accounted for 88.9%, mixed calcium stones for 9.3%. and uric acid stones for 1.9% of patients with stone formations after RYGB [9].

## Conclusions

In summary, this registry study of 58,366 patients undergoing primary RYGB, SG, or BPD-DS demonstrates a sixfold higher incidence for kidney stones in the surgical group compared to 583,660 controls from the normal population. Risk factors were higher age, hypertension and T2D at baseline, and in particular previous kidney stones. In contrast to other studies, we could not show that SG had a lower incidence rate of kidney stones than RYGB. We believe that further research concerning differences in the pathophysiology of kidney stone formation is important as well as preventive modalities in bariatric patients.
